# Intentional Binding Is Driven by the Mere Presence of an Action and Not by Motor Prediction

**DOI:** 10.1371/journal.pone.0029557

**Published:** 2012-01-17

**Authors:** Andrea Desantis, Gethin Hughes, Florian Waszak

**Affiliations:** 1 Université Paris Descartes, Sorbonne Paris Cité, Paris, France; 2 CNRS (Laboratoire Psychologie de la Perception, UMR 8158), Paris, France; 3 Ecole des Hautes Etudes en Sciences Sociales, Paris, France; 4 CNRS (Institut Jean Nicod UMR 8129), Paris, France; Royal Holloway, University of London, United Kingdom

## Abstract

Intentional binding refers to the fact that when a voluntary action produces a sensory outcome, action and outcome are perceived as being closer together in time. This phenomenon is often attributed, at least partially, to predictive motor mechanisms. However, previous studies failed to unequivocally attribute intentional binding to these mechanisms, since the contrasts that have been used to demonstrate intentional binding covered not only one but two processes: temporal control and motor identity prediction. In the present study we aimed to isolate the respective role of each of these processes in the emergence of intentional binding of action-effects. The results show that motor identity prediction does not modulate intentional binding of action-effects. Our findings cast doubts on the assumption that intentional binding of action effects is linked to internal forward predictive process.

## Introduction

Intentional binding refers to the observation that when a voluntary action produces a sensory outcome, action and outcome are perceived as closer together in time [1]. Notably, stimuli triggered by voluntary actions are perceived as occurring earlier in time, and actions producing sensory outcomes are perceived as occurring later in time, relative to a baseline in which actions and stimuli occur alone. This phenomenon is often interpreted as related to the experience of agency [2], [3], since the temporal compression of the interval between actions and consequences may help individuals determine whether a sensory event was caused by them or not.

It has been proposed that both predictive and postdictive mechanisms are responsible for the emergence of intentional binding [4]–[6]. Recent research has begun to dissociate the two different aspects of intentional binding – namely, the shift in the perceived time of the action and the perceived time of the action effect – with respect to the relative contributions of predictive and postdictive mechanisms. Notably Moore et al. [7] demonstrated that a disruption of pre-SMA – normally associated with predictive mechanisms [8] – by transcranial magnetic stimulation (TMS) affected the perception of the time of the sensory effect but not of the movement. This finding suggests that the shift of the perception of the action effect results from predictive processes, whereas the shift of the movement doesn't (but see [6]). Since in the present study we were interested in investigating the role of predictive action mechanisms in intentional binding, we chose to focus only on the perceived time of the action-effect.

It has been suggested that the predictive mechanisms underlying intentional binding are motor-based predictive processes [2], [6], [7], [9], [10]. According to a widely accepted theory of motor control, internal forward models predict the perceptual consequences that a given action produces [11]. It is assumed that the prediction provided by internal forward models can be very specific and pertain to the exact identity of the action's perceptual consequences. The motor prediction of the identity of the action perceptual consequence is used to provide internal feedback of the predicted outcome of an action which can be used before sensory feedback is available [11], thereby shifting earlier in time the perceived occurrence of action effects. However, the precise mechanism of this temporal shift remains unknown [12], [13].

However, more importantly, if predictive forward mechanisms drive binding, then it should only occur in situations in which the agent is able to predict the identity of the sensory event s/he is going to produce, for example, when a particular action triggers a particular tone (or when two particular actions trigger two particular tones). The present study aimed to directly assess the role of such motor identity prediction mechanisms in intentional binding and compare these to other processes such as temporal prediction and temporal control [14]. To illustrate these two mechanisms, the agent can temporally predict the occurrence of a stimulus, for example, when it is preceded by a cue at a fixed time before the stimulus, or when it is generated by a passive movement. In both situations participants would be able to predict the point in time at which the stimulus appears. The agent can temporally control the occurrence of a stimulus when s/he actually produces the stimulus by means of an action. In this instance participants can not only predict the onset of the stimulus, they also have control over the onset. Note that being able to predict/control the timing of a stimulus may alter a number of processes important for the perception of the stimulus. For example, temporal prediction may alter the allocation of attentional resources after the appearance of the cue, as highlighted by research in the field of temporal attention [15], [16]. Temporal control may simply allow participants to focus attention and reduce distraction even before triggering the stimulus event.

Intentional binding has normally been assessed by comparing an experimental condition in which a sensory event followed a voluntary action (which involved both temporal control and motor identity prediction) to a control condition in which the same sensory event followed either another stimulus [17]–[20] or the participant's passive movement provoked by the experimenter [1], [5], [9], [10], [21], [22]. For instance, Haggard, Clark and Kalogeras [1], asked participants to trigger a tone by executing a key-press at a time of their choice (experimental condition). Participants had to judge, in separate blocks, the time of either their key-press or the subsequent tone, by referring to a clock-hand that rotated around a clock face. They observed a perceptual shift of both the time of the action and the time of the sensory event, such that the two events were bound closer in time. Critically, these perceptual shifts did not occur when TMS induced the participant to make an involuntary key-press (control condition). In another recent study of Humphreys and Buehner [18] participants executed voluntary key-presses which resulted in the delivery of tone (experimental condition). In the control condition participants did not perform any actions; instead, they were presented with an audible click, which was followed by the same tone as in the experimental condition. Participants had to estimate the length of the interval between their button press and the tone (experimental condition), or the click and the tone (control condition). The authors found that action intervals were judged shorter than equivalent control intervals. In each of these experiments it is not possible to disentangle the role of motor identity prediction and temporal control since participants always performed a single action which leads to the same action effect, thus the experimental task typically included both motor identity prediction and temporal control while the control task included neither.

Thus, the aim of the present study was to investigate whether binding effects are modulated by motor identity prediction and therefore to determine whether motor predictive mechanisms contribute to the binding phenomenon. In a first experiment we contrasted a condition in which participants temporally controlled the onset of an auditory stimulus but could not predict its identity with a condition in which participants' actions generated specific auditory stimuli, thus allowing them to predict the exact identity of the auditory stimulus they were going to produce. If intentional binding is produced by motor identity prediction then it should be greater for the condition in which the identity of the effect is predicted by the chosen action. In a second experiment participants' action produced an auditory stimulus that was either congruent or incongruent with respect to the action-effect association they learned in a previous acquisition phase. If intentional binding is dependent on accurate prediction of the identity of the action effect then it should be greater in the congruent condition compared to the incongruent condition.

To foreshadow the results, our findings cast doubt on the assumption that intentional binding of action-effects is based on specific predictive motor mechanisms.

## Materials and Methods

### 1. Experiment 1

The purpose of Experiment 1 was to investigate the role that temporal control and motor identity prediction play in intentional binding of action-effects. Participants completed three experimental conditions. In the motor-identity and temporal-control conditions, they performed randomly left or right key-presses. In the motor-identity condition each key-press produced a specific auditory stimulus, thus allowing participants to predict the exact identity of the auditory effect. In the temporal control condition left/right key-press generated randomly one of two auditory stimuli. Participants were therefore unable to predict the exact identity of the sensory effect, but they nevertheless temporally controlled its onset. Finally, in the temporal-prediction condition auditory stimuli were externally generated and were preceded by a cue (a sound) at a fixed time before the target stimulus, thus making the target stimuli temporally predictable.

The comparison between the temporal-control and the temporal-prediction condition assesses the influence of temporal control on intentional binding of action-effects. The comparison between the temporal-control and motor-identity conditions assesses the influence of motor identity prediction. For example, if intentional binding is entirely due to internal forward models participants should manifest stronger anticipation in the motor-identity condition than in the two other conditions (with no difference between the latter). If, however, intentional binding is entirely due to temporal control participants should manifest stronger anticipation in the motor-identity condition and the temporal control condition (with no difference between these conditions) than in the temporal prediction condition. Of course, if both these factors are effective, the design also allows for the assessment of their relative influences.

#### 1.1. Participants

Twenty-four subjects (average age 25.12 years; sd = 5.11) participated in the Experiment 1 for an allowance of € 10/h. All had normal or corrected-to-normal vision, normal hearing and were naïve as to the hypothesis under investigation. They all gave written informed consent before participating in the experiment. The study was conducted in accordance with the Declaration of Helsinki and was approved by the Ethics Committee for Biomedical Research (CERB) Ile de France II.

#### 1.2. Material

Stimulus presentation and data acquisition were conducted using the Psychophysics Toolbox extensions [23], [24] for Matlab 7.5.0 running on a PC computer connected to a 19-in. 85 Hz CRT monitor (IIYAMA HM 903 DT A).

#### 1.3. Stimuli and Procedure

Participants completed three conditions (*motor-identity*, *temporal-control* and *temporal-prediction*) in separate blocks. Each block consisted of 100 trials. Block presentation was counterbalanced across subjects. In all trials of each condition participants were presented with a clock-face marked with 5 ‘min’ intervals and a clock-hand (1.5 cm of length and 0.1 cm of width) rotating with a period of 2560 ms. On each trial the initial clock-hand position was randomly chosen.

In the *motor-identity* and *temporal-control* conditions participants were instructed to carry out left or right key-presses in random order and about equally often. Feedback of the proportion of right and left key-presses was provided every 25 trials. Participants were instructed to avoid responding in a stereotyped way, at a pre-decided clock time, or during the first half rotation of the clock-hand. Participants' actions produced two auditory stimuli. The first sound, a 750 Hz sawtooth sound, was presented simultaneously with the participants' key-press from its onset to its off-set. The second was either a 1000 Hz (high) or a 500 Hz (low) pure tone. The duration of both tones was 100 ms. They were presented 450 ms after the onset of the participants action. In the *motor-identity* condition participants' actions triggered a specific tone. For half of the participants, a right key-press produced a high-tone and a left key-press produced low-tone. For the other half of participants, the mapping was reversed. In contrast, in the *temporal-control* condition both actions could trigger either a high-tone or a low-tone with a probability of .5.

In the *temporal-prediction* condition both auditory stimuli (the sawtooth sound and the high/low tone) were externally generated. The sawtooth sound served as cue to predict the onset of the subsequent high/low tone. However, participants could neither control the time of occurrence of the high/low tone nor predict its identity (the probability of occurrence of each tone was set to .5). The onsets and durations of the sawtooth sound were individually yoked to the action production times recorded in the immediately previous action condition. However, if the participant started the experiment with the temporal-prediction condition we used key-press duration and onset values drawn from a normal distribution characterized by the mean and standard deviation calculated from the action production times of the two previous participants.

In each condition the clock-hand stopped at a random position 1–2 sec. after the high/low tone and then disappeared. Thereafter, participants reported, using a computer keypad, the onset-time of the high/low tone. They were encouraged to use the highest possible precision, and were not restricted to use the numbers marked on the clock-face.

In order to ensure that the participants paid attention to the identity (high/low) of the action-effect, the experiment included 12% of catch trials where participants indicated whether they heard a high or a low-tone. Catch trials occurred randomly and equally often on high and low-tone trials.

Each block was preceded by an acquisition phase, where participants learned action-effect (action conditions) and stimulus-stimulus (temporal-prediction condition) contingencies. Each acquisition phase consisted of 50 trials. In the motor-identity acquisition phase participants associated right and left actions with a specific tone (high or low). In the temporal-control acquisition phase participants executed right or left key-presses followed either by a high or a low-tone with a probability of .5. Finally, in the temporal-prediction acquisition phase participants learnt to temporally predict the occurrence of the high/low tone from a cue (the sawtooth sound).

### 2. Experiment 2

The aim of Experiment 2 was to rule out a possible confound between motor identity prediction and temporal control that might have been involved in Experiment 1 (see results Experiment 1). As such, in Experiment 2, participants' actions produced an auditory stimulus that was either congruent or incongruent with respect to the action-effect association they learned in a previous acquisition phase.

Experiment 2 comprised an action-to-tone and a tone-to-tone block. Each block was preceded by an acquisition phase during which associations between either two actions (left or right action in the action-to-tone block) or two sounds (sound A or B in the tone-to-tone block), on the one hand, and two auditory stimuli (high/low tone) were formed. During both action-to-tone and tone-to-tone blocks we assessed the influence of these associations on participants' temporal estimations of the onset of high/low tones. In both blocks participants were presented with congruent and incongruent trials in which the associations they learned in the previous acquisition phases between actions/sounds and the subsequent tone was either respected or violated.

Accordingly, the internal forward models theory of intentional binding would predict a stronger anticipation in congruent trials than in incongruent trials. This assumption is supported by a recent study on sensory suppression, a perceptual phenomenon that is likewise suggested to be based on predictive forward model mechanisms [25], [26]. For instance, in Cardoso-Leite et al. [12] study participants associated to left and right hand action Gabor patches of different orientations. In the test phase, participants were presented with near threshold Gabor patches whose orientations were either congruent or incongruent with their learned action-effect association. On half of the trials no patch was presented, and participants were required to determine on each trial whether a Gabor patch was presented or not. Perceptual sensitivity (d′) was consistently lower in the congruent condition. To check that these effects were caused by motor identity prediction, and not caused by identity prediction independent of action, the authors ran a control task. In this task participants pressed both buttons at the same time and heard a concurrent sound. The pitch of this sound predicted which of the two orientations would be presented. As with the previous task, participants first acquired the relevant mappings and were then tested in a visual detection task using congruent and incongruent trials. Interestingly, the authors did not find any difference in sensitivity for congruent or incongruent trials in this control task, confirming that identity prediction alone (i.e. non motor identity prediction) did not drive the difference in the action prediction condition.

Thus, similarly if intentional binding of action-effects is based on predictive motor processes we should expect to observe: firstly, a stronger anticipation in the action congruent trials than in the action incongruent trials, and secondly, no difference between congruent and incongruent trials in the tone-to-tone prediction task. This would confirm unambiguously that motor identity prediction and not identity prediction independent of action drives the difference in our action-to-tone prediction condition, thus corroborating the idea that predictive motor processes play a crucial role on the emergence of the intentional biding of action effects.

#### 2.1. Participants

Thirty-two subjects (average age 25.91 years; sd = 5.52) participated in the experiment for an allowance of € 10/h. All had normal or corrected-to-normal vision, normal hearing and were naïve as to the hypothesis under investigation. They all gave written informed consent before participating in the experiment. The study was conducted in accordance with the Declaration of Helsinki and was approved by the Ethics Committee for Biomedical Research (CERB) Ile de France II.

#### 2.2. Material

See Experiment 1

#### 2.3. Stimuli and procedure

Participants completed an action-to-tone and a tone-to-tone condition. Condition presentation was counterbalanced across participants. Each condition included an acquisition phase and a test phase. During the acquisition phases an association was formed between two particular tones and either two actions (action acquisition phase) or two sounds (tone-to-tone acquisition phase). The test phases assessed the influence of these associations on the temporal perception of the tones. In both conditions participants were presented with the same clock-like stimulus we used in the Experiment 1.

Acquisition phases. Each acquisition phase consisted of 100 trials. In the *action-to-tone* acquisition phase participants executed left or right key-presses in a random order and about equally often. Feedback of the proportion of right and left key-presses was provided after 34 trials and 68 trials. They were instructed to avoid responding in a stereotyped way or during the first half rotation of the clock-hand. The initial clock-hand position was randomly chosen in each trial. For half of the participants, the right key-press produced a 1000 Hz pure tone (high-tone) and the left key-press produced a 500 Hz pure tone (low-tone). For the other half, the reverse mapping was used. The high/low tone lasted 100 ms and its onset was fixed to 400 ms after the participants' action onset.

In the *tone-to-tone* acquisition phase participants' actions were replaced with two sounds: a bell shaped sound and a sinusoidal sound (from now on labeled sound A and B, respectively), to which we applied a mid-level expansion using Adobe Audition version 1.0. The mean frequency of both sounds was set to 750 Hz and their duration was fixed to 200 ms. For half of the participants sound A and sound B were followed (after 400 ms SOA) by a high-tone and a low-tone, respectively. For the other half, the reverse mapping was used.

Although, previous studies on the intentional binding used an action/tone - effect interval of 200–300 ms [1], we decided to use a 400 ms SOA to give participants enough time to process the cue and to ensure that they could easily predict the identity of the subsequent tone. We were confident to be able to reproduce intentional binding with this SOA, indeed several recent studies observed binding with interval longer than 400 ms [10], [27]–[29].

The onset of sound A and B were individually yoked to the movement production times recorded in the action-to-tone acquisition phase. However, if the participant started the experiment with the tone-to-tone condition, the onset of sound A/B was yoked to the action production times of the previous participant.

Test phases. Both *action-to-tone* and *tone-to-tone* test phases consisted of 240 trials divided in 24 mini-blocks of 10 trials each. In the *action-to-tone* mini-blocks participants generated a high/low tone by performing left or right-key presses. During each mini-block they were required to execute either only left or only right key-press, resulting in 12 right and 12 left action-to-tone mini-blocks presented alternately and counterbalanced across subjects. Information concerning the action they had to execute was provided at the beginning of each mini-block.

In the *tone-to-tone* mini-blocks participants were presented with two externally generated auditory stimuli: a sound A or B and a subsequent high/low-tone. During each mini-block participant heard always the same first sound, resulting in 12 sound A and 12 sound B mini-blocks presented alternately with mini-blocks presentation being counterbalanced across subjects. Information concerning which first sound (A or B) they were going to hear was provided at the beginning of each mini-block.

In both action-to-tone and tone-to-tone mini-blocks participants were presented with congruent and incongruent trials in which the associations they learned in the previous acquisition phases between left/right action (action-to-tone condition) or sound A/B (tone-to-tone condition), and the subsequent tone was respected or violated, respectively.

We chose to block the trials in to mini-blocks to simplify the task for the participants and to maximize their ability to predict the identity of the effect tone. Including a random ordering of trials in the tone-to-tone condition would likely have been particularly confusing for participants, since they would first have to identify the first tone and use that to predict the second tone. By blocking both tasks we ensured that in each condition participants could easily predict the effect-tone, and that this would be reinforced on each trial by the presentation of the paired tone or action. This increased predictability should also help to highlight the incongruent trials in both tasks. The probability of the participant being presented with an incongruent tone was set to .3 for 8 mini-blocks and to .2 and .1 for the remaining two groups of 8 mini-blocks, resulting in an overall probability of to .2 for both action-to-tone and tone-to-tone condition. We chose this probability distribution in order to prevent participants from understanding the pattern of presentation of the incongruent tones. Incongruent trials were randomly distributed between the 4^th^ and the last trial of each mini-block.

In each trial the clock-hand stopped at a random position 1–2 sec. after the high/low tone and then disappeared. Thereafter, as in the Experiment 1, participants reported the onset of the high/low tone. We included 15% of catch trials where participants indicated whether they heard a high or a low-tone. Catch trials were randomly distributed and occurred equally often on congruent and incongruent trials.

## Results

### 1. Experiment 1

Participants made on average 52.12% of their voluntary actions with their right-hand and 47.88% with their left-hand in the temporal control condition and 51.5% right-hand action and 48.5% left-hand action in the motor-identity condition, indicating a non-significant tendency to press with the right hand in both conditions, t(23) = 1.925, p = .0666 and t(23) = 1.5698 = .1301, respectively.

The mean temporal estimation error, defined as the difference between estimated and actual onset of the tone was calculated for each condition (Estimation error = Onset(estimated)−onset(physical)).

**Figure 1:**
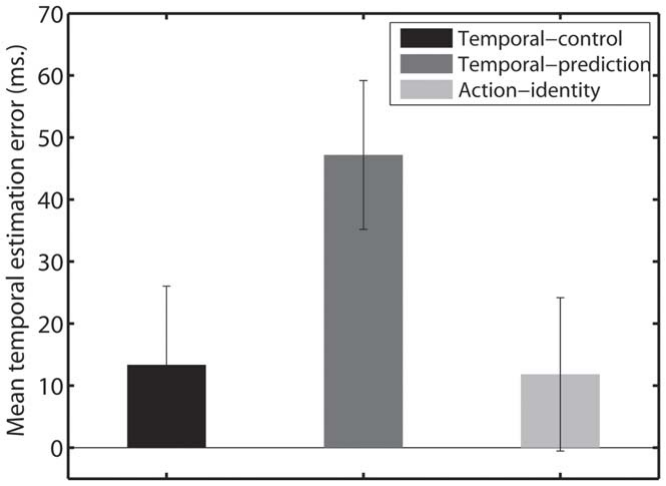
Mean temporal estimation error in ms for the temporal-control, temporal-prediction and motor-identity condition. Bars represent standard errors.

A repeated measures analysis of variance (ANOVA) with Condition (temporal control, temporal-prediction and motor-identity) and Tone (high and low) as factors revealed a significant main effect of condition on participants' temporal estimations error, F(2, 46) = 14.909, p = .0000. Neither the main effect of tone nor its interaction with Condition was significant. Paired two-tailed t-test revealed a significant difference between temporal-control and temporal-prediction, t(23) = 4.193, p = .0003, with the tones being significantly anticipated in the temporal-control compared to the temporal-prediction condition. The tones were also significantly anticipated in the motor-identity condition compared to the temporal-prediction condition t(23) = 5.7615, p = .0000. However, no significant difference was found between temporal-control and motor-identity condition, t(23) = 0.2042, p = .8399 (Fig. 1).

Catch trial analysis revealed that participants made about 99% of correct response in the three conditions. A repeated measure ANOVA showed that responses to catch trials did not differed between conditions F(2, 46) = .10698, p = .89877. Thus, it is unlikely that the absence of any modulations of identity prediction on subjects' estimations is due to a lack of attention of the tones that participants generated.

#### Preliminary discussion

To sum up, we observed the classic intentional binding effect as usually assessed by comparing an action condition (or rather two action conditions in our case) to a temporal-prediction condition. However, the prediction of the identity of the action's perceptual consequence did not influence participants' temporal estimations at all. This suggests the temporal control of the onset of the action-effect is sufficient to drive the binding we observed in both action conditions.

However, one may argue that motor identity prediction was also involved in the temporal-control condition. Namely, in a context where two action-effects have the same probability of occurrence, the motor systems might be able to predict both sensory consequences. Indeed, the prediction of more than one action perceptual consequence might be important given that it potentially allows an organism to rapidly correct and reorient its behavior. To rule out this possibility we ran a second experiment in which participants' actions produced an auditory stimulus that was either congruent or incongruent with respect to the action-effect association they learned in a previous acquisition phase (see above).

### 2. Experiment 2

Catch trial analysis revealed that one participant had to be excluded from further analysis due to extremely poor performance (the subject correctly identified only 29.17% of catch tones during the action-to-tone catch trials and 62.5% during the tone-to-tone catch trials). For all the other participants, performance was very high for both conditions (mean(action-to-tone) = 98.85% of correct responses; mean(tone-to-tone) = 96.03% of correct responses). Error rates did not differ between the conditions.

The mean temporal estimation error, defined as the difference between estimated and actual onset of the tone was calculated for each condition. Anticipatory estimates are represented as negative values. A repeated measures analysis of variance (ANOVA) with Condition (action-to-tone and tone-to-tone) and Congruency (congruent and incongruent) as factors revealed a significant main effect of condition on participants' temporal estimations error, F(1, 30) = 51.360, p = .0000, with tones being significantly anticipated in the action-to-tone compared to the tone-to-tone condition. However the interaction between Condition and Congruency was not significant F(1, 30) = 1.4839, p = .2326.

**Figure 2:**
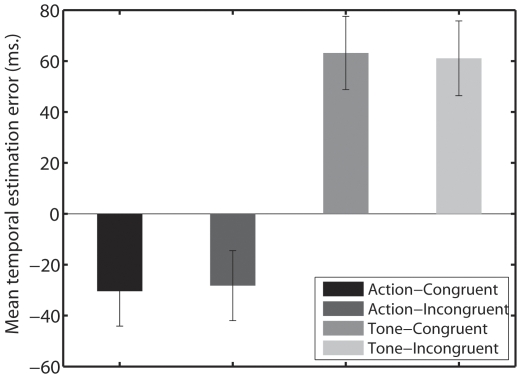
Mean temporal estimation error in ms for both action-to-tone and tone-to-tone blocks. Bars represent standard errors.

Further two paired two-tailed t-tests showed no congruency effect neither in the action-to-tone condition nor in the tone-to-tone condition, t(30) = 0.9868, p = .3316 and t(30) = 0.9355, p = .3569 respectively (Fig. 2), suggesting that the prediction of the identity of the auditory stimulus does not play any role in the estimation of its onset time.

#### Preliminary discussion

Thus, as in the experiment 1 we replicated the classical intentional binding effect as usually assessed by comparing an action-to-tone condition (action-to-tone blocks) to a temporal-prediction condition (tone-to-tone blocks). However, we did not find any effect of identity prediction in either the action-to-tone condition or the tone-to-tone condition. This corroborates the conclusions of Experiment 1, namely that motor identity prediction does not influence intentional binding.

## Discussion

The aim of the present study was to assess the role of predictive motor mechanisms on the emergence of intentional binding. Although intentional binding has often been explained in terms of predictive motor processes [2], [6], [7], [9], [10], [19], previous studies did not unambiguously show motor identity prediction to be its basis, as they compared conditions that differed in terms of both temporal control and motor identity prediction processes.

To assess the influence of predictive motor processes on intentional binding we ran two experiments including contrasts differing in terms motor identity prediction only. In both experiments we replicated the classical binding effect with auditory stimuli being perceived earlier when they were produced by the participants' actions compared to when they were externally generated. However, we found that neither the ability to predict the precise identity of the stimulus dependent on which action was performed (Experiment 1), nor the accuracy of this prediction (Experiment 2) influenced the magnitude of the binding effect. These findings suggest that intentional binding of action-effects is not based on predictive motor processes.

We observed significant differences between conditions that varied in the presence or absence of temporal control, but no such differences associated with the presence of motor identity prediction. We defined temporal control as the ability to control (via an action) the onset of the relevant stimulus (action-effect). How might such temporal control produce the binding effect? One possibility is that although the action-effect in the two conditions were matched in terms of overall temporal prediction, processing of the cue might differ between the two conditions leading to differences in allocation of attentional resources. More precisely, the cue (the action in the temporal-control condition or the auditory cue in the temporal-prediction condition) would only be predictable in the temporal-control condition since participants can decide when to press the button while in the temporal prediction condition they would not know when exactly the auditory cue would appear. This may in itself produce an orienting response to the auditory cue, that is absent in the temporal-control condition and that could, in turn, influence sensory processing of the subsequent stimulus. Indeed, the capacity to decide when to execute an action (temporal-control condition) may have allowed participants to reduce distraction during each trial and to focus attention to the subsequent stimulus, leading them to perceive the action-effect as occurring earlier due to prior entry [30], [31].

One might argue that the absence of any congruency effect in the second experiment might be due to the fact that participants failed to associate action-effect pairs in the acquisition phase. Fifty repetitions of a given action-effect coupling might simply have been insufficient to link the motor code with the subsequent sensory effect. A failure to associate action and effect in the acquisition phase would make action effect prediction in the test phase impossible. Indeed, some previous studies that investigated action-effect learning and action effect prediction included an acquisition phase comprising approximately 100 trials of a given action-effect association [32]–[35]. However, more recent studies showed that action-effect associations are learned rather quickly, namely, after much less than 50 repetitions of an action-effect pairing [36], [37]. Wolfensteller & Ruge [31] showed that even 8 repetitions are sufficient to link an action to a subsequent effect. We therefore feel confident that the participants of Experiment 2 did learn the action-effect associations.

Another possible objection is that participants might have linked their actions to the incongruent tone in the test phase, after which they begin to predict both tones for both actions. However, several findings make this unlikely. Firstly, Wolfensteller & Ruge [31] showed that although action-effect associations are learned already after few encounters, the associations get more stable as the number of repetitions increases. Secondly, Elsner and Hommel [33] showed that the frequency of co-occurrence of an action and an effect is also a critical factor for the acquisition of action-effect learning. Learning in the test phase is, thus, unlikely. Finally, similarly to our experiment, recent studies successfully manipulated the congruency of a given sensory effect with respect to the association that participants learned in a previous acquisition phase. They showed that the congruency of action and predicted effect modulate motor control related phenomena, such as sensory attenuation of self-generated stimuli [12] and deviance processing in the brain [30].

A further objection might be that by blocking the response hand in the second experiment this might have put the participants in a less predictive “set” compared to a condition in which the response hand is chosen by the participants. Indeed, it has been shown spatial attention effects are larger when attention is cued on a trial-by-trial basis than when attention is constant for a whole block [38], suggesting that employing mini-blocks rather than allowing the prediction to vary on a trial-to-trial basis may have reduced the magnitude of any prediction.

However, as we pointed out in the procedure section, blocking trials in to mini-blocks simplified the task for the participants. Including a random ordering of trials in the tone-to-tone condition would likely have been particularly confusing for participants, since they would first have to identify the first tone and use that to predict the second tone. Additionally, blocking the trials should help to highlight the incongruent trials in both tasks.

Intentional binding has often been interpreted as related to the sense of agency, since it may help individuals determine whether a sensory event was caused by them or not [1]. This interpretation is due in part to the fact that binding has reliably been observed in cases in which individuals are the agent of an action, thus when internal efferent information is provided by motor processes that are used to prepare and execute an action [2]. However, our results seem to suggest that binding is not linked to motor predictive mechanism but rather to temporal control. Do these findings weaken the link between intentional binding and the sense of agency? A definitive answer to this question would be premature at this stage. Further studies need to be carried out in order to clearly highlight the relation between binding and the sense of agency. However, even if the phenomenon of binding of actions to their effects is not due to motor predictive processes it could still contribute to the emergence of sense of agency by, for instance, accentuating people's perception of the temporal contiguity between actions and their effects.

The results we report here provide new insights into mechanisms of intentional binding of action-effects. They cast doubt on the assumption that intentional binding is based on predictive motor mechanisms. Instead, the temporal control of a stimulus, by means of a voluntary action, might be sufficient to trigger the binding effect observed in the present and previous experiments.
